# Short- and Long-Term Outcomes of Laparoscopic Versus Open Resection for Gastric Gastrointestinal Stromal Tumors: A Propensity Score-Matching Analysis

**DOI:** 10.1097/MD.0000000000003135

**Published:** 2016-04-18

**Authors:** Qing-Feng Chen, Chang-Ming Huang, Mi Lin, Jian-Xian Lin, Jun Lu, Chao-Hui Zheng, Ping Li, Jian-Wei Xie, Jia-Bin Wang, Qi-Yue Chen, Long-Long Cao, Ru-Hong Tu

**Affiliations:** From the Department of Gastric Surgery, Fujian Medical University Union Hospital, Fuzhou, Fujian Province, China.

## Abstract

Published reports on laparoscopic resection of gastric gastrointestinal stromal tumor (GIST) were limited to small experiences and selection bias.

Two hundred fourteen patients who underwent primary gastric GIST resection at our institution (January 2006–December 2012) were identified from a prospectively collected database. Laparoscopic resections (LAP) were performed in 133 patients, and open resections (OPEN) were performed in 81 patients. The short- and long-term outcomes were analyzed using propensity-score matching (PSM) by comparing the clinicopathological factors between these groups.

The tumor resection method and tumor size were significantly different between the LAP and OPEN groups. After PSM, there were no differences (*P* > 0.05) in these clinicopathological factors. The LAP group had less blood loss and shorter operation time, time to first flatus, time to first fluid diet, time to gastric tube removal, and postoperative stay before PSM. In addition, there were no differences regarding the time of drainage tube removal or hospitalization expense. Other than the time of gastric tube removal, which was similar in these 2 groups, the short-term outcomes were similar before and after PSM. The rates of postoperative complications in the LAP and OPEN groups were 6.8% and 22.8%, respectively, before PSM (*P* = 0.001) and 5.6% and 22.5%, respectively, after PSM (*P* = 0.004). The multivariate analyses for complications showed that tumors were located in the middle of the stomach, and the operation method and proximal gastrectomy were independent risk factors before and after PSM. The 5-year cumulative survival rates in the LAP and OPEN groups were 95.4% and 85.9%, respectively, (*P* = 0.07) before PSM and 93.1% and 91.9%, respectively, (*P* = 0.69) after PSM (not significantly different).

Laparoscopic resection for gastric GISTs had better short-term outcomes and similar long-term outcomes compared with open surgery. Localized gastric GISTs can be treated with laparoscopic surgery.

## INTRODUCTION

Gastrointestinal stromal tumors (GISTs) are the most common types of mesenchymal tumors of the gastrointestinal tract, and these tumors occur most frequently in the stomach (50%–70%).^1–3^ Complete resection is the primary treatment for gastric GIST. Most gastric GISTs are localized in the submucosa and grow distensibly. These tumors infrequently invade nearby lymph nodes.^4^ These unique biological characteristics provide favorable conditions for laparoscopic surgery. Since Lukaszczyk et al^5^ performed laparoscopic surgery for gastric stromal tumors for the first time in 1992, the safety and feasibility of the laparoscopic resection of gastric GISTs have been confirmed by several studies.^6–10^ However, selection bias (tumor size and location) may have been present in these previous studies. In addition, there is a lack of randomized controlled studies and reports on the long-term outcomes of laparoscopic gastric resection for gastric GIST. In nonrandomized controlled trials, propensity-score matching (PSM),^11^ which calculates the propensity score for each patient by the logistic regression model and analyze the matched-pair data, can control for selection bias. Therefore, this study summarizes the clinicopathological data for 214 patients who underwent resection of primary gastric GISTs at our institution from January 2006 to December 2014. PSM was used to investigate the short- and long-term effects of laparoscopic and open gastric resection for GISTs.

## METHODS

### Study Population

**FIGURE 1:**
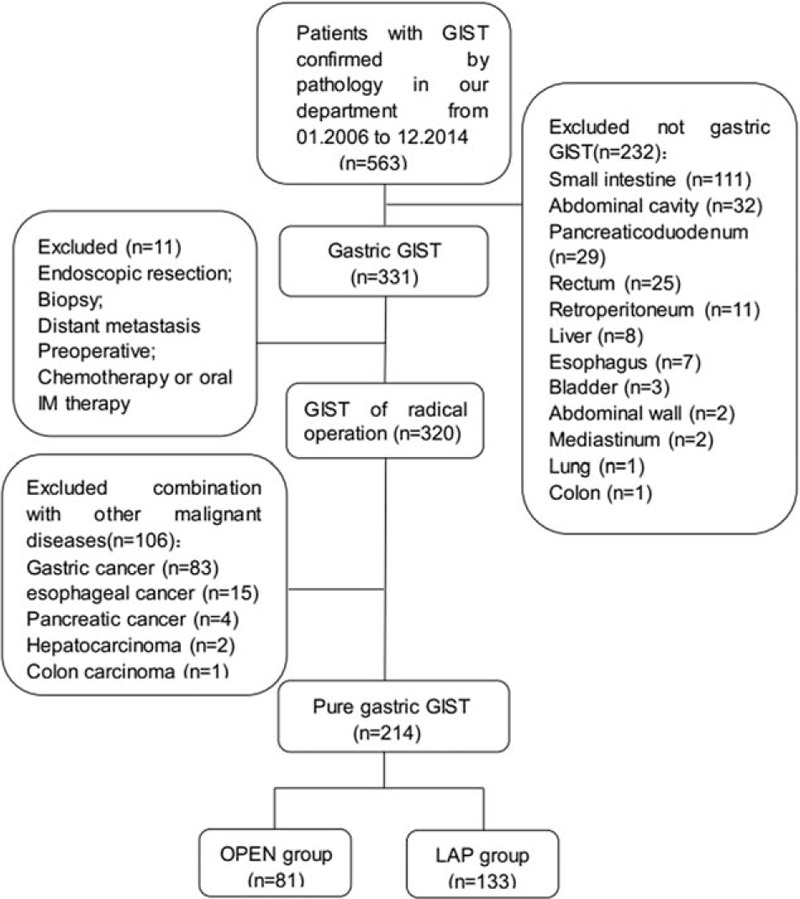
Enrollment of patients in the study.

The study cohort consisted of 563 GIST (confirmed by pathology) patients at the Fujian Medical University Union Hospital from January 2006 to December 2014. The inclusion criteria were as follows: primary gastric GIST (confirmed by pathology) patients and R0 resection. The exclusion criteria were as follows: (1) GISTs originating outside the stomach, (2) treated with neither laparoscopic nor open surgery, (3) received preoperative chemotherapy or oral imatinib (IM) treatment, (4) distant metastasis, and (5) combined with other malignant diseases (confirmed by pathology). Finally, a total of 214 cases were included in the study, which consisted of laparoscopic resection for gastric GIST in 133 cases (LAP group) and open surgery in 81 cases (OPEN group) (Figure 1).

### Variables and Definitions

Body mass index (BMI) ≥25 was identified as overweight according to WHO criteria. Gastric GIST was graded by the modified National Institutes of Health (NIH) risk classification scheme.^12^ The mitotic rates were defined as the number of mitoses per 50 high-power fields. The tumor size was defined as the maximum tumor diameter. Blood loss was quantified via the operation or anesthesia records. Preoperative comorbidities were classified according to the Charlson comorbidity index (CCI), and postoperative complications were classified according to the Clavien-Dindo Classification scheme.^13^

### Surgery Procedures

The surgery types consisted of laparoscopic and open resection for gastric GISTs, and the tumor resection types were wedge resection, proximal gastrectomy, distal gastrectomy, and total gastrectomy.

### Follow-Up

Specially trained researchers used outpatient records, visitation, letters, and telephone calls to follow up with the patients after the operation. The last follow-up period was March 2015. The follow-up information included survival status, postoperative review results, tumor recurrence, and (or) metastasis and adjuvant therapy. The survival time was calculated as the time from diagnosis to the last contact, the date of death, or the date that the survival information was collected.

### Statistical Analyses

All statistical analyses were performed using the SPSS 18.0 statistical software. The propensity score for each patient was calculated by a multiple factor logistic regression model, and we imposed a caliper of 0.20 of the standard deviation of the logit of the propensity score. According to the nearest neighbor matching principle and the nonreplacement principle (which indicates that a single case cannot be selected multiple times), we matched participants using a simple 1:1 matching. The measurement data are presented as the means ± standard deviations. Categorical data were compared with a χ^2^ test or Fisher exact test. The variables with *P* < 0.1 in the univariate analysis were subsequently included in a multivariate binary logistic regression model. The results of the univariate and multivariate analyses were expressed as odds ratios (ORs) with corresponding 95% confidence intervals (95% CIs). The survival rates were calculated using the Kaplan-Meier method, which used the log-rank test to detect differences in the survival curves of the various subgroups. *P* < 0.05 was considered significant.

## RESULTS

### Clinicopathological Characteristics

All of the patients’ tumor margins were negative, and there was no intraoperative tumor rupture. LAP patients were more likely to undergo gastric wedge resection and have smaller tumors compared with the open group (*P* < 0.05). We set nine indices as matching covariant variables for PSM, including age, sex, body mass index (BMI), tumor size, mitotic rate, modified NIH risk classification, tumor resection type, tumor location, and IM treatment after surgery. Finally, 71 patient pairs were enrolled in this study. There were no significant differences (*P* > 0.05) in tumor size, mitotic rate, modified NIH risk classification, tumor resection methods, or IM treatment after surgery between these groups after PSM (Table 1).

**TABLE 1:**
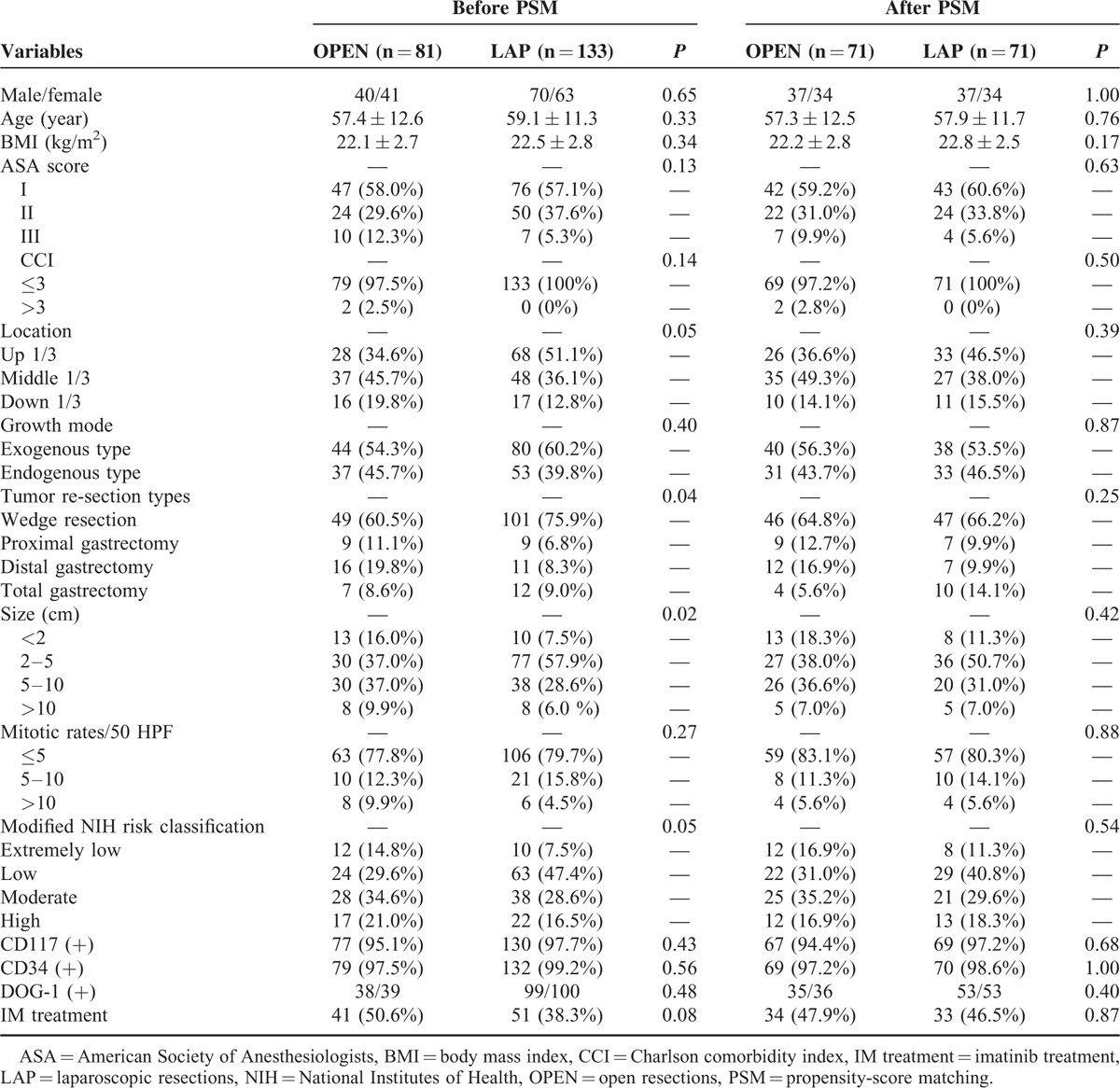
Baseline Characteristics of Eligible Patients Before and After PSM

### Perioperative and Postoperative Results

Before PSM and compared with the OPEN group, the LAP group had less blood loss, shorter operation time, time to first flatus, time to first fluid diet, time to gastric tube removal, and postoperative stay. The 2 groups did not differ in the drainage tube removal times or hospitalization expenses. The short-term outcomes were similar before and after PSM, other than the time of gastric tube removal, which was similar in these 2 groups after PSM (Table 2).

**TABLE 2:**
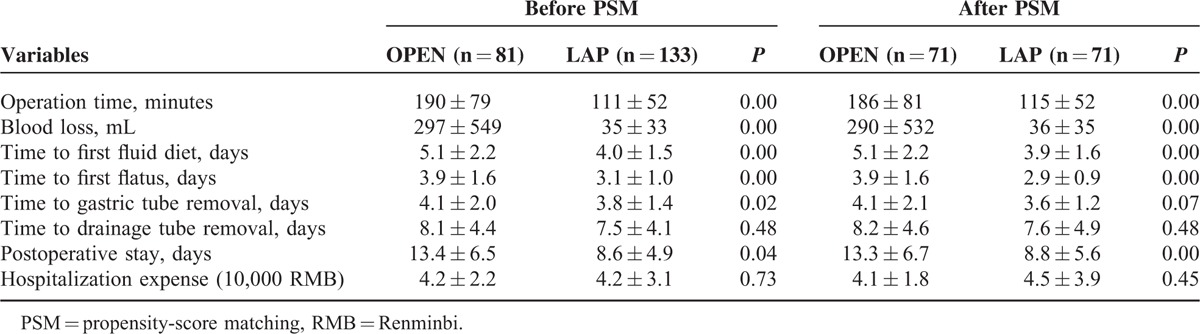
Perioperative and Postoperative Results

### Postoperative Complications

The postoperative complications were categorized according to the Clavien-Dindo Classification scheme. The postoperative complication rates in the LAP and OPEN groups were 6.8% and 22.8%, respectively, before PSM (*P* = 0.001) and 5.6% and 22.5%, respectively, after PSM (*P* = 0.004). The percentages of category I-II complications were 4.5% and 19.8%, respectively, before PSM (*P* = 0.001) and 2.8% and 21.1%, respectively, after PSM (*P* = 0.001). There were no differences in the percentages of category III-IV complications between the LAP and OPEN groups, which were (2.3% and 2.5% before PSM and 1.4% and 2.8% after PSM, respectively) (Table 3).

**TABLE 3:**
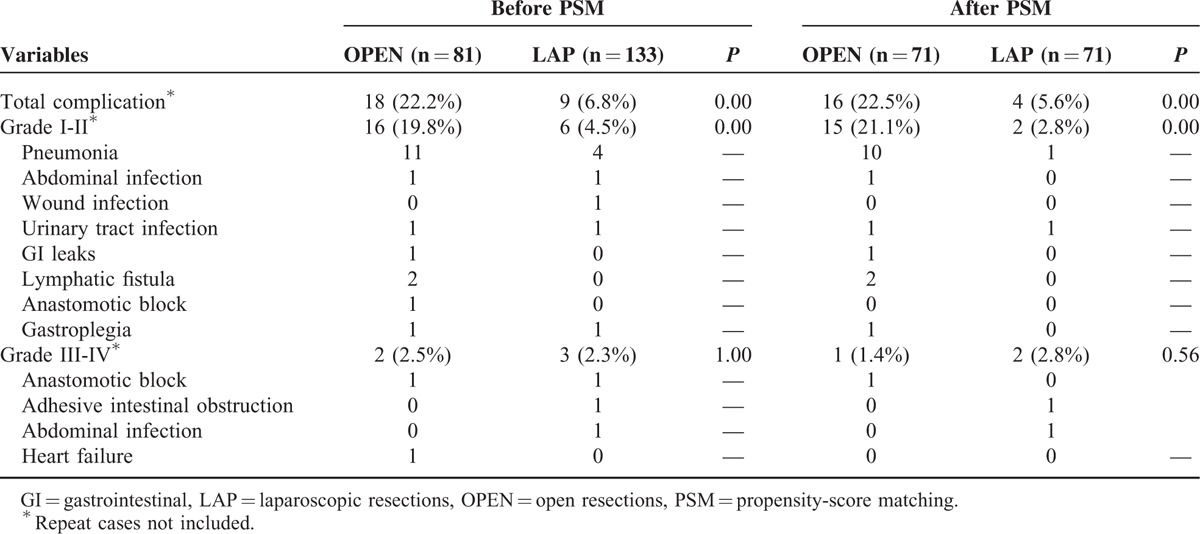
Postoperative Complications Before and After PSM

### Univariate and Multivariate Analyses of Postoperative Complications

A univariate analysis showed that tumor location in the middle third part of the stomach, laparoscopic surgery, and proximal gastric resection were close related to the postoperative complications before and after PSM. A further multivariate analysis showed that in terms of postoperative complications, laparoscopic surgery (OR = 0.27, 0.08–0.92) was a protective factor, while tumor location in the middle third part of the stomach (OR = 9.43, 1.87–47.59) and proximal gastric resection (OR = 6.82, 1.07–43.30) were independent risk factors (Tables 4 and 5).

**TABLE 4:**
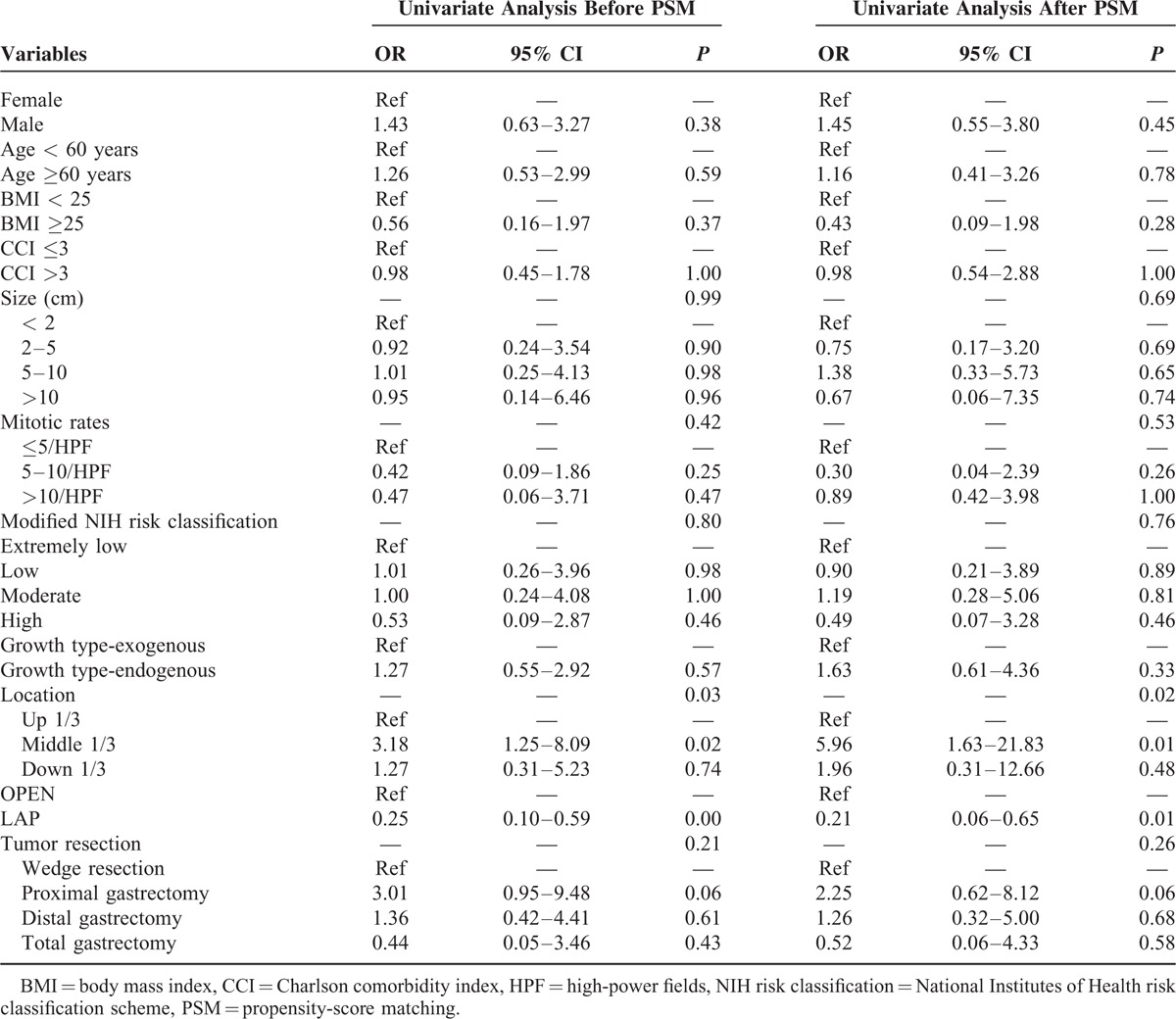
Univariate Analysis of Postoperative Complications

**TABLE 5:**
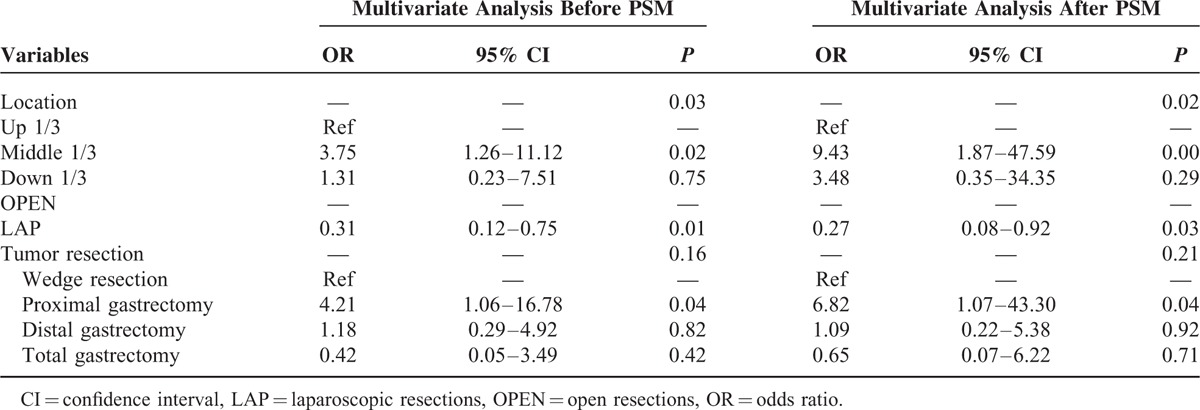
Multivariate Analysis of Postoperative Complications

### Long-Term Outcomes

A total of 200 patients (93.5%) were followed up before PSM. The median follow-up time was 35 months (range 1–111 months). And 132 patients (93.0%) were followed up after PSM, and the median follow-up time was 36 months (range 1–111 months). The 3-year cumulative survival rates of the LAP and OPEN groups were 98.0% and 91.0%, respectively (*P* = 0.056). The 5-year cumulative survival rates of the LAP and OPEN groups were 95.4% and 85.9%, respectively, (*P* = 0.07) before PSM. However, after PSM, there were no differences in the 3- or 5-year cumulative survival rates between the LAP and OPEN groups (93.1% and 95.6%, *P* = 0.35; 93.1% and 91.9%, *P* = 0.69, respectively).

**FIGURE 2:**
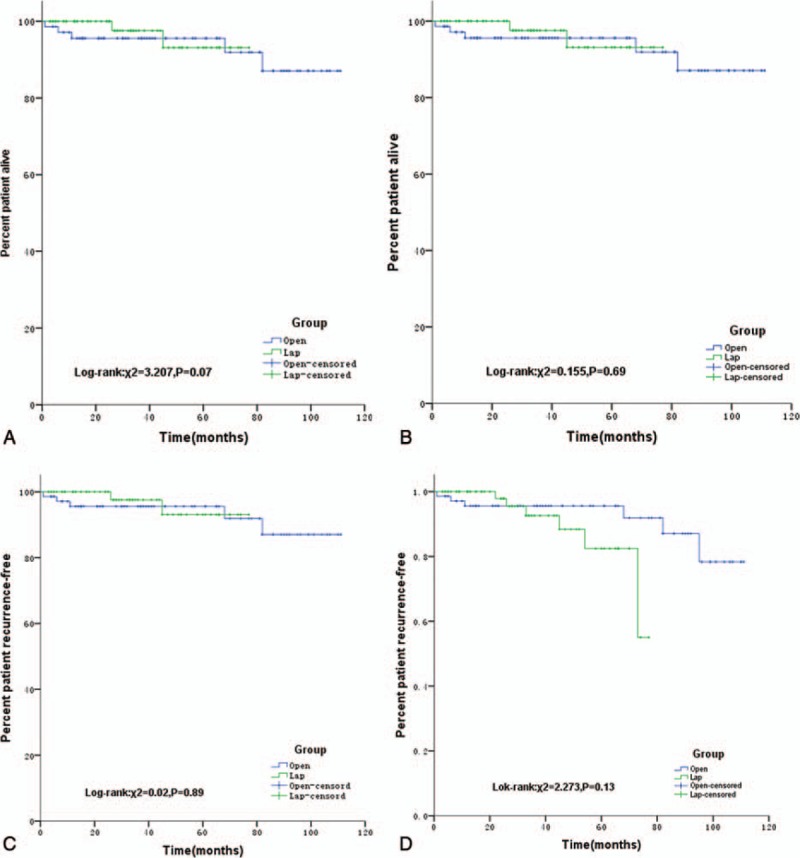
A, Kaplan-Meier curves for OS between the LAP group and OPEN group before PSM (χ^2^ = 3.207, *P* = 0.07). B, Kaplan-Meier curves for OS between the LAP group and OPEN group after PSM (χ^2^ = 0.155, *P* = 0.69). C, Kaplan-Meier curves for PFS between the LAP group and OPEN group before PSM (χ^2^ = 0.02, *P* = 0.89). D, Kaplan-Meier curves for PFS between the LAP group and OPEN group after PSM (χ^2^ = 2.273, *P* = 0.13). LAP = laparoscopic resections, OPEN = open resections, OS = overall survival, PFS = progress free survival, PSM = propensity-score matching.

Before PSM, recurrence occurred in 9 (6.8%) and 11 cases (13.6%) in the LAP and OPEN groups, respectively. After PSM, recurrence occurred in 6 (8.5%) and 5 cases (7.0%) in the LAP and OPEN groups, respectively. These rates were not significantly different. Before PSM, the 3-year recurrence-free survival rates of the LAP and OPEN groups were 93.4% and 91.0%, respectively (*P* = 0.44). Before PSM, the 5-year recurrence-free survival rates of the LAP and OPEN groups were 82.2% and 86.1%, respectively (*P* = 0.89). After PSM, the 3-year recurrence-free survival rates for the LAP and OPEN groups were 92.6% and 95.6%, respectively (*P* = 0.88). After PSM, the 5-year recurrence-free survival rates were 82.5% and 91.9%, respectively (*P* = 0.13) (Figure 2).

## DISCUSSION

Surgery is the initial treatment for localized or potentially removable GISTs, and the surgical options are open or laparoscopic surgery. However, the choice of surgery method is influenced by several factors. For relatively young patients with healthy body conditions, smaller tumors or lower risks, laparoscopic operations are more likely to be performed.^6,8,9,14,15^ Therefore, these studies were conducted with a selection bias that decreases the comparability of these results and affects the results of the research. For example, in the study of Goh et al,^6^ the tumor size in the laparoscopic group was smaller than in the open group (3.1 cm vs 4.5 cm, *P* = 0.043). The very low to low risk cases in the open group accounted for 43% of the cases, and this proportion was 60% in the laparoscopic group. While Bischof et al^9^ controlled for selection bias by PSM, the data still contained differences between the resection methods and tumor sizes (Table 6). In this study, the patients whose tumor was small and located in the upper third of the stomach were more likely to receive laparoscopic surgery, and laparoscopic surgery was associated with a higher proportion of the use of wedge resection. These differences could bias the results. In retrospective studies, PSM^16–18^ could have balanced the confounding variables, and the results would have been similar to randomized controlled studies. PSM can greatly increase the comparability between groups. Therefore, this study used the PSM method to control for selection bias, tumor size, mitotic rate, risk classification, tumor location, resection method, and other clinicopathological characteristics to account for significant differences.

**TABLE 6:**
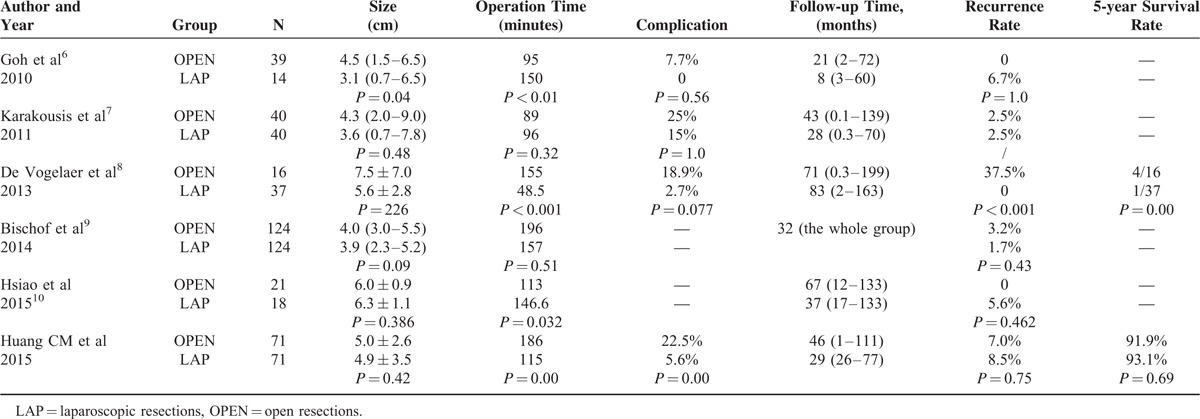
Journals Reviewed

Historically, gastric GISTs were initially treated by open surgery. However, open surgery induces greater trauma for patients, and those patients recover slowly after surgery. Laparoscopic surgery has many advantages compared to open surgery, and these advantages have been confirmed by several randomized controlled trials for gastric cancer, colon cancer, and nephrectomy.^20–22^ In addition, laparoscopic surgery is being gradually used for the treatment of GIST. Scholars initially believed that the difficulty of laparoscopic surgery would increase when tumors were large or located in the lesser curvature, cardiac or pylorus regions. Furthermore, laparoscopic surgery could increase the risk of tumor rupture. In 2012, the European Society for Medical Oncology clearly stated that laparoscopic surgery is not recommended for large tumors.^19^ However, with the improvements of laparoscopic technology, the majority of researchers currently considered laparoscopic surgery to be a safe and feasible treatment for gastric GISTs when the potential complications are well managed. In 2014, the National Comprehensive Cancer Network (NCCN) guidelines^20^ also modified the old version of the guidelines regarding the tumor size limitation for laparoscopic surgery. In this study, the tumor sizes of the LAP group were 1 to 11.3 cm, and laparoscopic surgery was successfully performed for all of the tumors without any ruptures. To avoid tumor rupture, we attempted not to touch the tumor directly. Tissue separation, capture, anastomoses were performed in the normal gastric tissue surrounding the tumor. In addition, the tumor was removed from abdominal cavity with a specimen bag.

This study showed that the LAP group performed significantly better than the OPEN group regarding operation time, blood loss, and recovery of gastrointestinal function before and after PSM. Thus, laparoscopic operations for GISTs resulted in better short-term outcomes than open surgery. These results are consistent with previous literature reports. In addition, the results showed that laparoscopic surgery can reduce the incidence of postoperative complications, especially grade I-II complications. Wan et al^21^ compared 68 cases of laparoscopic surgery and 88 cases of open surgery, and the rates of postoperative complications were 5.9% and 22.7% in the LAP and OPEN groups, respectively (*P* = 0.004). The study conducted by Bischof et al^9^ also showed that the incidence of complications (more severe than grade III) after laparoscopic surgery was significantly lower than open surgery. In this study, the most common complication in the open group was pulmonary infection. The open operation results in greater trauma, a longer operation time, and a longer time until gastric tube removal. Furthermore, the postoperative digestive tract function recovery is slow. These factors may contribute to the increased rate of lung infections. We recommend that the operation times should be decreased as much as possible during open surgeries, and clinicians should monitor for pulmonary infections in patients with long times to gastric tube removal. Using a logistic regression analysis, we observed that laparoscopic surgery is a protective factor (OR = 0.27) for postoperative complications. Thus, laparoscopic surgery is safe and feasible for gastric GIST patients. In addition, proximal gastric resection (OR = 9.43) and tumors located in the middle third of the stomach (OR = 6.82) were independent risk factors for postoperative complications. The higher postoperative complication rates may result from the wide tumor separation ranges and more complex digestive tract reconstructions that are required during proximal gastrectomy. However, those patients whose tumor located in the gastroesophageal junction or pylorus often require more difficult excisions as well as more complicated reconstructions. Unfortunately, such cases occupied only a small portion in total patients included in this study. Therefore, whether the conclusion that tumor location of middle third of the stomach is an independent risk factor of postoperative complications is correct remains to be discussed. And a further large-scale or prospective clinical study about this issue will follow. We also found that these 2 groups did not differ in the hospitalization expenses. We think the reason is that the cost of laparoscopic surgery was higher than open surgery, but the operation time of open surgery was longer, and the incidence of complications was higher, which increased the hospitalization expenses of open surgery.

The prognosis of patients with gastric GIST was initially poor, and the 5-year survival rate was only 42%.^22^ Recently, the prognosis of patients with GIST has improved significantly due to the improvement of surgical techniques and the clinical application of the molecularly targeted drug IM.^23–25^ In this study, the 5-year cumulative survival rates of the LAP and OPEN groups were 95.4% and 85.9%, respectively, before PSM. Thus, laparoscopic surgery has a better prognosis trend, although the *P* value was not statistically significant, which could have been due to the differences in the clinical and pathological characteristics of the 2 groups. After the selection biases of these 2 groups were balanced by the PSM method, the 5-year cumulative survival rates of the LAP and OPEN groups were 93.1% and 91.9, respectively, and the rates were not statistically different. The long-term outcomes of laparoscopic and open surgery were similar, which was consistent with previous reports (Table 6), but larger sample sizes and prospective, multicenter randomized studies are still required to provide more accurate evidence.

In summary, laparoscopic resection for gastric GIST resulted in improved short-term outcomes and similar long-term outcomes compared with open surgery. Localized gastric GIST can be treated by laparoscopic surgery. In this study, there were differences in tumor location and size between the 2 groups before PSM. Although the significance of the results did not change obviously by the application of the PSM method, we believed that PSM could balance the confounding variables and the results would be closer to those from randomized controlled studies, which would greatly increase the comparability between the 2 groups. Thus, the effects of surgery methods on postoperative outcomes of GIST patients could be illustrated more objectively and exactly. Although this is a retrospective study, it can provide references for the subsequent randomized clinical studies.
